# Geometry-independent antenna based on Epsilon-near-zero medium

**DOI:** 10.1038/s41467-022-31013-z

**Published:** 2022-06-22

**Authors:** Hao Li, Ziheng Zhou, Yijing He, Wangyu Sun, Yue Li, Iñigo Liberal, Nader Engheta

**Affiliations:** 1 Department of Electronic Engineering, Beijing National Research Center for Information Science and Technology, Tsinghua University, Beijing, 100084 China; 2 Department of Electrical and Electronic Engineering, Public University of Navarre, Pamplona, 31006 Spain; 3 Department of Electrical and Systems Engineering, University of Pennsylvania, Philadelphia, PA 19104 USA

**Keywords:** Electrical and electronic engineering, Metamaterials, Metamaterials, Nanophotonics and plasmonics

## Abstract

It is well known that electromagnetic radiation from radiating elements (e.g., antennas, apertures, etc.) shows dependence on the element’s geometry shape in terms of operating frequencies. This basic principle is ubiquitous in the design of radiators in multiple applications spanning from microwave, to optics and plasmonics. The emergence of epsilon-near-zero media exceptionally allows for an infinite wavelength of electromagnetic waves, manifesting exotic spatially-static wave dynamics which is not dependent on geometry. In this work, we analyze theoretically and verify experimentally such geometry-independent features for radiation, thus presenting a novel class of radiating resonators, i.e., antennas, with an operating frequency irrelevant to the geometry shape while only determined by the host material’s dispersions. Despite being translated into different shapes and topologies, the designed epsilon-near-zero antenna resonates at a same frequency, while exhibiting very different far-field radiation patterns, with beams varying from wide to narrow, or even from single to multiple. Additionally, the photonic doping technique is employed to facilitate the high-efficiency radiation. The material-determined geometry-independent radiation may lead to numerous applications in flexible design and manufacturing for wireless communications, sensing, and wavefront engineering.

## Abstract (web summary)

This paper proposes an unconventional type of antennas that are composed by epsilon-near-zero (ENZ) media. The operating frequencies of such antennas are independent of their geometries, and are determined by material dispersion.

## Introduction

Radiation of electromagnetic fields has been a fundamental topic in physics and engineering for many decades, and has led to essential applications in a variety of fields such as wireless communications^1^, remote sensing^2,3^, wireless power transmission^4,5^, to name a few. A resonator with coupling to the free space (such as an open cavity) can leak the confined field at the resonance to the radiation wave outside. Such a process is a phenomenon where the geometry feature (i.e., size and shape) determines the frequency feature. This dependence can be equivalently understood from the perspectives of the eigenmodes of the radiating resonator, where the resonance frequency is usually determined by its size and geometry^6^. A well-known example is the dipole antenna^7–9^, widely adopted in microwave and nano-optics, whose operating frequency is directly related to the length of its arms. The Fabry-Perot cavity^10,11^, as another familiar case, resonates and generates laser only if its length is an integer multiple of half the wavelength in the medium.

Since such “geometry-dependence” is pervasive in radiation phenomena, radiating cavity resonators with operating frequency independent to the geometry, if exist, would represent a qualitatively different class of radiators. It would introduce valuable degrees of freedom for tailoring the far-field radiation pattern of a resonator via controlling its geometry or the spatial distribution of radiating apertures, while maintaining the operating (resonant) frequency unchanged. This is contrary to our usual intuition in wave dynamics, where the spatial distribution of the electromagnetic mode of a resonator is described by the wavelength λ in the medium, which in turn is related to the oscillation frequency f of electromagnetic fields by the fundamental constraint *fλ* = c/*n* where n is the refractive index of the medium filling that resonator. To break the limit of the minimum size of a resonant cavity to be half a wavelength, researchers have proposed an optical cavity based on hyperbolic metamaterials and achieve a size-independent resonance in a miniaturized geometry^12^. Due to its small size and the large wave numbers in hyperbolic metamaterials, the cavity does not naturally work as an efficient radiator. Therefore, realizing such geometry-independent radiator is still challenging.

However, recent years have witnessed the exiting development of the field of near-zero-index (NZI) media^13,14^. Depending on which material’s constitutive parameter is close to zero, NZI media are categorized as epsilon-near-zero (ENZ)^15^, mu-near-zero (MNZ)^16^, and epsilon-and-mu-near-zero (EMNZ)^17^ media. The electromagnetic wave in the NZI media features a stretched wavelength, therefore exhibiting some exotic spatially static wave dynamics, i.e., effectively decoupling the space and time. The NZI media have induced a number of unusual wave phenomena and functionalities. Among them are supercoupling of energy through arbitrarily-shaped channels^18–20^, wavefront transformation^21–26^, boosting optical nonlinearity^27,28^, trapping light in open structures^29^, and novel quantum effects, just to name a few. Moreover, the technique of photonic doping^30–37^ of ENZ media provides additional convenience to control the NZI effect, leading to applications in on-chip devices^36^, general impedance matching^37^, etc. In all these applications, the operating frequency is determined by the dispersion of the material, in particular, the frequency at which the ENZ property is achieved rather than the geometry shapes.

In this work, we demonstrate that NZI media open up an opportunity to pursue the resonant frequency of electromagnetic radiation with independence to geometry shapes of the radiators. Inspired by the ENZ medium with suppressed spatial variation of fields^37^, we devise a deformable geometry-invariant ENZ antenna. Actually, the possibility of achieving certain geometry-independent antennas has been shown, as reported by using specific zeroth-order resonant mode, as an effective ENZ mode, to obtain a size-invariant frequency along *one dimension*^38,39^. However, geometry invariance along one-dimension is strictly constrained to length invariance, and access to higher dimensionalities is required to fully take advantage of geometry-invariant effects. To this end, here we put forward a generalized concept of geometry-independent antennas based on two-dimensional (2D) ENZ materials rather than a specific mode. In this manner, maintaining an unchanged frequency is an intrinsic feature that applies to arbitrarily shaped 2D ENZ materials beyond those particular effective ENZ resonances to break the geometric restriction of antennas. Different from the geometry-determined operating frequency of a conventional antenna design relying on electromagnetic resonance, this ENZ antenna exhibits a stable operating frequency which is unchanged under transformations of its geometry and only depends on the material’s dispersive constitutive parameters. This material-determined radiating property discovers a completely different working mechanism for antennas and offers a new approach for antenna design. On the other hand, the wavefront as well as the spatial power distribution of the radiation can be designed according to the shape of the ENZ cavity and the aperture arrangement. To attain a high-efficiency radiation, we embed a photonic dopant^33^ into the ENZ medium, thus optimizing the impedance matching from the source to the free space. An equivalent circuit model is established in order to quantitatively illustrate the underlying mechanism of the ENZ cavity antenna. These results are firstly theoretically and numerically analyzed, then we launch the experiment as a proof-of-concept to demonstrate the deformation performance of the antenna using specific examples where three ENZ antennas are designed with different cross-sectional shapes with the assist of waveguide-emulated plasmonic materials^40,41^. Both numerical and experimental results reveal that the antenna’s operating frequency is not changed under the process of geometry transformation from one case to another, with varying beam orientations and widths. The concept of ENZ-based geometry-independent antenna provides a novel route to shape the spatial radiation pattern without any change in frequency, thus yielding applications such as programmable beam generation and the flexible wavefront engineering via geometry transformation.

## Results

### Concept of ENZ-based radiating cavities

**Fig. 1:**
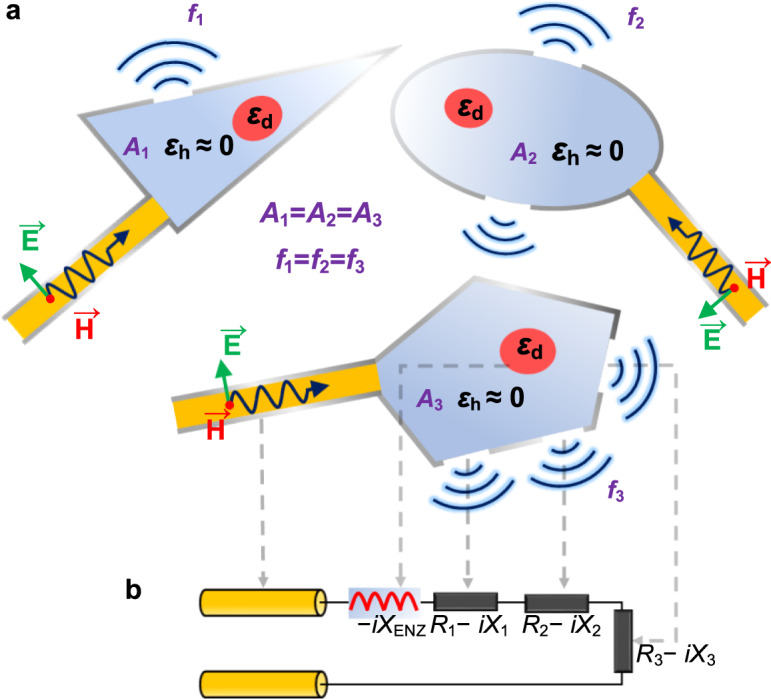
General idea of geometry-independent antenna. **a** Sketch of ENZ antennas with arbitrary cross-sectional shapes supporting geometry-independent modes. With their different geometries, the radiation directions of the antennas change from case to case, while the frequency is kept the same. **b** Equivalent lumped-circuit model of the proposed structure, which is based on an ENZ cavity fed by a waveguide.

The general concept of our proposed idea is shown in Fig. 1a. With the deformation of the ENZ radiator, the frequency of the radiating fields is kept the same while the wavefront and the radiation directions are efficiently adjusted. As shown in Fig. 1a, the radiation is based on an arbitrary shaped cavity filled with a photonically-doped ENZ medium. As demonstrated in ref. ^37^, a closed ENZ cavity containing a dielectric inclusion supports eigenmodes with a geometry-invariant resonant frequency. We are now demonstrating that this phenomenon can be also observed in ENZ cavities with open boundaries, serving as a geometry-invariant radiator of electromagnetic waves. As sketched in Fig. 1a, we consider a 2D ENZ region partially enclosed by perfect electric conductor (PEC) walls deformed into arbitrary shapes but with a same cross-sectional area *A*. The ENZ medium has a permittivity described by Drude model that *ε*_h_(*f*) = 1 − *f*_p_^2^/*f* ^2^ and is doped with a dielectric rod inclusion whose cross section is shaped as a circle or a square. As demonstrated in ref. ^33^, this medium is equivalent to a homogeneous ENZ medium with an effective permeability *μ*_eff_, in which the magnetic field *H*_0_ is uniform over the ENZ region. Several radiation aperture slots are etched on the PEC wall and a parallel plate waveguide with a thickness of *h* is used to excite the ENZ radiator. Applying Faraday’s law along the boundary of the ENZ cavity leads to:

1 $${\int }_{\partial A}{{{{{\boldsymbol{E}}}}}}\cdot d{{{{{\boldsymbol{l}}}}}}=i\omega {\mu }_{{{{{{\rm{eff}}}}}}}A{H}_{0}$$

In full PEC-enclosed cavities discussed in ref. ^34^, the tangential electric field is zero at every point along the boundary, so the integration of the electric fields along the boundary is trivially zero. However, in this radiating ENZ cavity, the electric field circulation is contributed by the electric fields at the port and all the slots, which does not equal zero. In order to describe the relations between the electric and magnetic fields on these 2D apertures, we define a 2D surface impedance by the integral of electric field (i.e., voltage) over the aperture divided by the magnetic field at the aperture, as $Z={\int }_{{L}_{n}}{{{{{\boldsymbol{E}}}}}}\cdot d{{{{{\boldsymbol{l}}}}}}/{H}_{0}={{{{{\rm{V}}}}}}/{H}_{0}$. Here L_n_ is along the n-th radiating aperture. Under this definition, the n-th radiating aperture with a length of *l*_n_ is modelled as an impedance boundary on which the field distribution satisfies that *Z*_n_ = *V*_n_/*H*_0_. Here *Z*_n_ has a complex value, which can be written as *Z*_n_ = *R*_n_-*iX*_n_. A detailed discussion on the numerical value of *Z*_n_ is provided in the Supplementary Note 1. For the parallel-plate waveguide, the characteristic impedance is also defined in a 2D manner by the incident voltage *V*_inc_ divided by the incident magnetic field *H*_inc_. Since the thickness of the waveguide is h, the 2D characteristic impedance of the waveguide is *Z*_0_= *hE*_inc_/*H*_inc_. A similar equation also holds for the reflected ones that *Z*_0_ = *hE*_r_/*H*_r_. Considering both the incident and reflected waves, the fields on the ports are *H*_0_= *H*_inc_ − *H*_r_ and *E*= *E*_inc_ + *E*_r_. By applying these expressions to Eq. (2), it is derived that

2 $${Z}_{0}\left({H}_{{{{{{\rm{inc}}}}}}}+{H}_{r}\right)+{\sum }_{n}{Z}_{n}{H}_{0}-i\omega {\mu }_{{{{{{\rm{eff}}}}}}}A{H}_{0}=0$$

Equation (2) can be alternatively explained as the Kirchhoff’s voltage law by modelling the 2D ENZ cavity as a series lumped-circuit, which is depicted in Fig. 1(b). In this lumped loop, the current is characterized by the surface current density, which numerically equals to the magnetic field on the surface. As a result, the previously defined 2D surface impedance also equals the ratio of voltage over the surface current density, which is defined here as the 2D impedance of the structure in Fig. 1(a) (extending infinitely in the out-of-plane axis). In the following part, all the terms “current” and “impedance” (including resistance, reactance, inductance, and capacitance) refer to the surface current density and 2D impedance as defined above. It is worth mentioning that under this definition, the loop current and impedance have units of A/m and Ωˑm, respectively. In addition, each aperture is modelled as a radiating load with a 2D impedance of *Z*_*n*_ = *R*_*n*_-*iX*_*n*_ in the circuit. The feeding waveguide is represented by a transmission line with a source at the other terminal and the 2D characteristic impedance is *Z*_0_. As reported in ref. ^36^, the doped ENZ medium with closed boundary is modelled as a series inductor or capacitor depending on its effective permeability. The contribution of the ENZ host is equivalent to a lump element to the loop with a 2D reactance of

3 $${X}_{{ENZ}}=\omega {\mu }_{{{{{{\rm{eff}}}}}}}A$$

For *μ*_eff_ > 0, the doped ENZ medium behaves as an inductor with a 2D inductance *L* = *X*_ENZ_/*ω* = *μ*_eff_ *A*. Here the 2D inductance *L* has a unit of H·m. While *μ*_eff_ < 0, the ENZ host performs like a capacitor rather than an inductor.

When the mutual couplings between apertures are small enough to be ignored, it can be derived from Eqs. (2) and (3) that the reflection coefficient seen at the feeding waveguide looking into the ENZ cavity is calculated to be

4 $$R=\frac{{H}_{r}}{{H}_{{inc}}}=\frac{{\sum }_{n}\left({R}_{n}-i{X}_{n}\right)-i\omega {\mu }_{{{{{{\rm{eff}}}}}}}A-{Z}_{0}}{{\sum }_{n}\left({R}_{n}-i{X}_{n}\right)-i\omega {\mu }_{{{{{{\rm{eff}}}}}}}A+{Z}_{0}}$$

This important parameter describes how much power is reflected back to the source and is related to how much power radiates into free space, thus depicting the radiation efficiency at this frequency. A critical issue is the antenna’s impedance matching, which ensures that the impinging power is totally radiated to the free space rather than reflected back. To minimize the reflection coefficient *R* at the input port, the area and effective permeability of the ENZ host are supposed to satisfy that ${\sum }_{n}\left(-i{X}_{n}\right)-i\omega {\mu }_{{{{{{\rm{eff}}}}}}}A=0$ (i.e., a zero reactance of the antenna) and the 2D characteristic impedance of the waveguide should be ${Z}_{0}={\sum }_{n}{R}_{n}$ (i.e., the real part of the impedance must be matched). In fact, an undoped ENZ medium with μ_eff_ = 1 introduces a large inductance, which blocks the input power from efficient radiation. To solve this problem, the photonic doping method is used as reported in refs. ^30,35^, where the relative permeability μ_eff_ of the whole ENZ media is dependent on the geometry and the permittivity of the dielectric dopant together with the total area of the ENZ host. Therefore, by carefully tuning the dopant’s material and size, we can maximize the power injected into the ENZ radiator at a selected frequency and the radiating waves are strong enough to be observed.

A more important property indicated by Eq. (4) is that the reflection coefficient is not influenced by the geometry shape of the antenna, indicating that deformation of the antenna does not disturb the impedance matching and the resonant frequency. According to Eq. (4), the antenna’s resonant frequency is calculated to be ${\omega }_{0}=-({\sum }_{n}{X}_{n})/({\mu }_{{{{{{\rm{eff}}}}}}}A)$. By the impedance matching method above, it can be tuned to be exactly *ω*_0_ = *ω*_p_, thus exciting a resonance at the plasma frequency of the ENZ host and most of the input powers are radiated into the free space. As shown in the equation above, the resonant frequency is only related to the constitutive parameters of the material and the aperture’s radiating impedance, while the specific shape and the positions of the apertures have no impacts on it. In other words, the antenna can be deformed into any shape but maintaining an unchanged operating frequency which is fixed at *ω*_p_ of the host material. This unique property rarely exists in a conventional antenna design, which has an operating frequency influenced by its shape. This geometry-independence inspires a new counter-intuitive property of geometry-flexibility for the antenna based on ENZ medium. A conventional antenna usually has a definite shape which cannot be easily changed once designed. However, this geometry-independent ENZ antenna is able to be transformed into arbitrary shapes as long as the total cross section is kept invariant. This deformation enables a controllable far field radiation pattern of the antenna illustrated by the fact that the wavefront is conformal with the boundary of the radiating ENZ medium according to previous studies^20^. According to the Huygens’ principle^42^, the spatial distribution of radiation field, particularly the angular distribution, is determined by the shape of the wavefront. As a result, a completely different angular distribution of the electromagnetic energy flux of the same time-harmonic signal can be achieved simply by changing the shape of the ENZ radiator.

### Simulation and experimental verifications

**Fig. 2:**
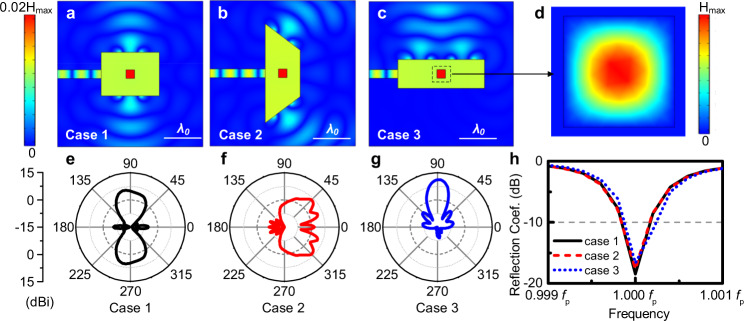
Numerical verifications of the shape-flexible properties of 2D structures. **a**–**c** Snapshots of the simulation results of magnetic fields of cases 1–3. The sizes of the rectangular ENZ hosts in case 1–3 are 1.54(1.15 λ_0_^2^ (80(60 mm^2^) and 2.31(0.77 λ_0_^2^ (120 × 40 mm^2^). In case 2, a trapezoid with their four sides being 2.62 λ_0_(136 mm), 1.15 λ_0_ (60 mm), 1.23 λ_0_(64 mm), and 1.15 λ_0_ (60 mm) is used. The slots, waveguides, and the dopants are the same in all configurations. **d** A time snapshot of the magnetic field inside the dopant. **e**–**g** Simulation results for the far-field radiation gain patterns of the antennas with different shapes and (**h**) the reflection coefficients for the three cases.

To validate the theoretical model discussed above, simulations on 2D doped ENZ antennas are launched using COMSOL Multiphysics® 5.5. For verification of the deformable performance of the antenna, as examples we take three specific shapes. As the first example, we consider a rectangular ENZ host with the permittivity described by the Drude model, i.e., *ε*_h_(*f*) = 1 − *f*_p_^2^/*f* ^2^ where *f*_p_ = 5.767 GHz. This antenna is transformed into three different shapes with a same total area of 1.77 *λ*_p_^2^ (λ_p_ is the free-space wavelength at *f*_p_) doped with a dielectric impurity with a size of 0.227 × 0.227 *λ*_p_^2^. In the first and the third case, it is shaped to be rectangular while in the second case a trapezoid shape is obtained via deformation. The relative permittivity of the dopant is 9.9. In this case, the effective relative permeability of the ENZ media is 0.045 according to ref. ^36^. On the boundary of each ENZ medium, four 3 mm-wide slots are etched and placed along the boundary at either one or two sides. An air-filled parallel-plate waveguide with a thickness of *h* = 0.23 *λ*_p_ (12 mm) is used for feeding. The simulated magnetic field distributions and reflection coefficients are depicted in Fig. 2 from which it can be observed that the deformations of ENZ host and radiation slots do not affect the impedance matching. From Fig. 2a–c, the magnitudes of the magnetic field are depicted. An unchanged uniform distribution is found inside the ENZ medium while different spatial distributions are observed in the free space for the different shapes of the ENZ hosts and the positions of the apertures. The field inside the dopant is described as a TE^z^_11_ mode for the magnetic field distribution is sinusoidal along both x and y directions. As shown in Fig. 2h, the reflection coefficients at these three cases are shown within a frequency range from 0.999*f*_p_ to 1.001*f*_p_. Following Eq. (4) and the discussions above, the resonance frequency is fixed at *f*_p_ where the reflection coefficient is not changed by deformations. Moreover, the angular distributions of the radiated power are shown in Fig. 2e–g in which they are normalized to their averages with respect to angle, respectively. The changed radiation wavefronts and the unchanged internal waveforms together confirm that the frequency response of this antenna is determined by the material’s properties rather than the geometries.

It is worth mentioning that all the geometry-independent results are obtained when operating at the plasma frequency *f*_p_ where the permittivity of the host medium is zero. Actually, due to the finite quality (Q) factor, the impedance matching is achieved in a frequency range around *f*_p_, denoted as the impedance bandwidth. A parametric study of the Q factor based on both analytical and numerical methods are launched to investigate the ways to enhance the bandwidth and the geometry-invariant properties of a low-Q ENZ antenna (see Supplementary Note 2 and Supplementary figure 2 within the Supplementary Materials). It can be concluded that although we can achieve a wider impedance bandwidth, the geometry-invariant performance is only achieved at the plasma frequency *ω*_p_ because Eq. (4) does not hold for the frequency that *ω* ≠ *ω*_p_. Consequently, geometry deformations may cause deteriorations on impedance matching at frequencies other than *ω*=*ω*_p_. In other words, the bandwidth may be influenced by the specific geometry shape.

**Fig. 3:**
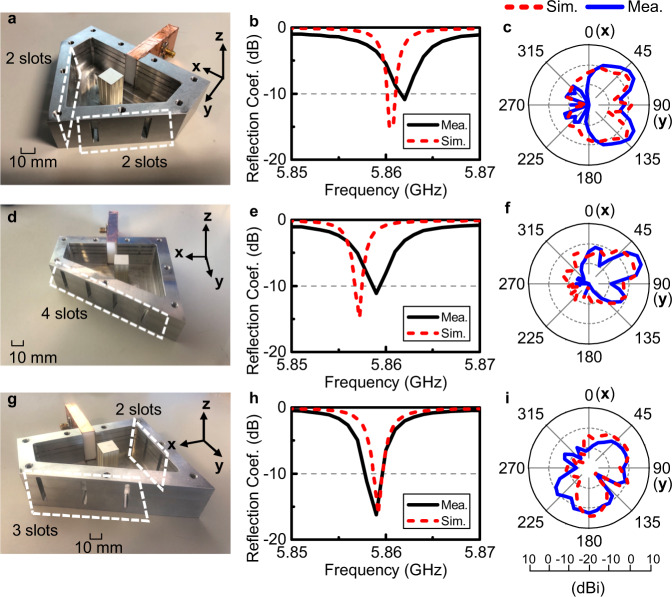
Experimental demonstrations of geometry-independent ENZ-based antennas. **a** Photograph of Antenna 1 where the metallic cover is not installed yet. **b** Simulated and measured reflection coefficients of Ant. 1. **c** The simulated and measured angular distributions of radiated fields of Ant. 1 in the E-plane which is parallel to the electric field. **d** Photograph of Antenna 2 where the metallic cover is not installed yet. **e** Simulated and measured reflection coefficients of Ant. 2. **f** The simulated and measured angular distributions of radiated fields of Ant. 2 in the E-plane which is parallel to the electric field. **g** Photograph of Antenna 3 where the metallic cover is not installed yet. **h** Simulated and measured reflection coefficients of Ant. 3. **i** The simulated and measured angular distributions of radiated fields of Ant. 3 in the E-plane which is parallel to the electric field.

To further validate this geometry-independent property, we experimentally test the impedance and radiation performances of ENZ antenna deformed into certain geometries and slot distributions. As demonstrated in ref. ^40^, a rectangular waveguide operating at its TE_10_ mode behaves effectively as a medium with a permittivity dispersion described by a lossless Drude model. In particular, the waveguide is equivalent to an ENZ medium when it is operating at its cutoff frequency. Based on this concept, we conducted a series of experiments to examine the deformation-immune performance of the antenna. For convenience, we did not use a flexible structure but designed and fabricated three different three-dimensional (3D) structures as representative experimental platforms instead, denoted as Antenna 1, 2, and 3 (Ant. 1–3). Each experimental platform is composed of a slotted metal cavity with a height of half wavelength emulating an ENZ medium, a dielectric block as the dopant, and a copper-coated Teflon brick as the feeding waveguide. The geometry configuration of each antenna is discussed in detail (see Supplementary figure 4 and the Supplementary Note 3) from which one can calculate that the cross section area of each resonator is 3200 mm^2^ and the volume is 83,200 mm^3^ and the fabricated prototypes are shown in the left column of Fig. 3 (Fig. 3a, d, g). In these panels, the metal covers are not assembled in order to show the inner structures clearly in the photographs, but they are assembled during the experiments. The slots etched on the boundaries are labelled using a white frame.

The reflection coefficients of all these prototypes are measured using a vector network analyzer and compared with the simulation results obtained via full-wave electromagnetic simulator CST Microwave Studio 2016. The results of these three prototypes are shown in the second column of Fig. 3, numbered as Fig. 3b, e, h. In spite of the shape differences, the resonance frequencies and reflection coefficients of Ant. 1 to 3 are all kept the same, demonstrating that the temporal behaviors, i.e., operating frequencies of the radiators are independent of the specific shapes of the ENZ media. Small frequency shifts in the resonance frequencies are observed and the fractional frequency shift is about 0.04%, which is a relatively small value. These shifts are mainly due to the higher order modes such as TE_30_ and TE_50_ modes generated by mode mismatching on the slots, which are not contained in the description of the structure as an ENZ medium. A comparison among the simulated results of Ant. 1 to 3 is depicted and it shows that the resonant frequencies are almost the same (see Supplementary figure 5 and the Supplementary Note 4). It is worth emphasizing that these three cases are only examples on which we conducted the experiment. Actually, the antenna is able to be reshaped into any geometries with an unchanged resonance frequency. The spatial distributions of the radiations are also experimentally tested by measuring the angular distributions of the radiated power in a standard microwave anechoic chamber. In the third column of Fig. 3, the simulated and measured gains of Ant. 1 to 3 are depicted. A comparison among the simulation results for the gains of Ant.1 to 3 are depicted in Supplementary fig. 5d–f in the Supplementary Material. The main beams are perpendicular to the radiation apertures of the ENZ hosts in these three cases as a result of the uniform magnitude and phase distributions of the magnetic field in ENZ media. To further verify this, the simulation results for the magnitudes of magnetic field distributed over the structures are shown and compared (see Supplementary fig. 5a–c in the Supplementary Material). These results all support the theoretical and numerical analysis.

### Applications in coded electromagnetic radiations and multi-functional antennas

**Fig. 4:**
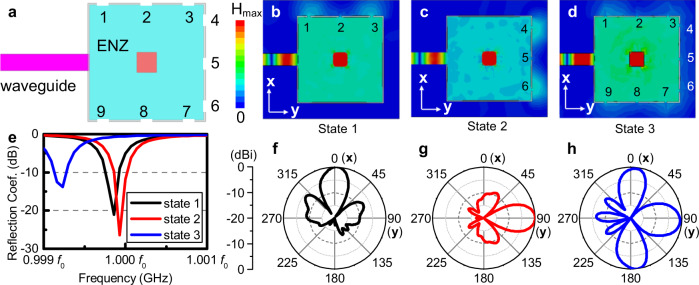
Antenna with coded radiated beam capabilities. **a** Sketch of the structure of a flexible ENZ antenna with coded radiated beam capabilities. State 1 is formed when slot 1,2, and 3 are turned “ON”, which corresponds to the code “111000000”. State 2 and State 3 correspond to codes “000111000” and “111111111”, respectively. **b**–**d** Colormap of the magnitude of the magnetic fields at the central plane of State 1–3. **e** The simulation results for reflection coefficients. **f**–**h** The normalized angular distributions of the radiated power in the xy-plane.

This geometry independent platform might provide inspiration for novel engineering applications. As is discussed in the previous sections, the positions of the radiation apertures have no impact on the frequency of the radiation field. However, when the apertures are moved along the boundary, the wavefront is reshaped and the angular distribution of radiated field is changed. Consider an ENZ cavity with *N* identical radiating apertures in which *n* apertures are open and others are closed. Each opening pattern is corresponding to a binary code where “0” represents that the aperture is closed and “1” represents that is open. By controlling the opening and closing of each aperture, the angular distributions of radiated powers can be encoded and all the states share the same frequency. A sketch of such beam encoder is depicted in Fig. 4. In Fig. 4a, a square ENZ cavity doped with a dielectric rod is depicted and 9 slots are etched on the boundary. Each slot is switched to either “ON” or “OFF” state where it serves as an opening aperture or a conducting boundary (see Supplementary fig. 6 and the Supplementary Note 5). In this case, each radiation is corresponded to a 9-bit state in which the *n*-th bit is “1”, i.e., the *n*-th slot is switched “ON”. Three typical states are discussed and simulated. For state “111000000”, the 1-st, 2-nd, and 3-rd slots are switched “ON” and the radiation is steered to *+x* direction. For the second one, the 4-th, 5-th, and 6-th slots are switched “ON” so that such radiation is encoded to be “00011100”. As a comparison, we also consider the state where all slots are switched “ON” and encode this state to be “111111111”. The simulation results for the magnetic field snapshots shown in Fig. 4b–d reveals that the magnetic field distribution in the ENZ host remains the same while the radiation patterns are tailored by selecting which slots to be switched “ON”. The unchanged reflections in Fig. 4e with a fractional frequency shift of 0.08% and steered radiation beams from a single one to multiple ones shown in Fig. 4f–h also show that the antenna is capable of generating beams in three different manners at a given frequency, with wavefronts and the radiation directions being flexibly changed. A relatively larger frequency shift is due to the larger radiation reactance when 9 slots are switched on than those when 3 slots are switched on. For more complicated cases, we have also investigated and present them in Supplementary Materials (see Supplementary fig. 7 and Supplementary Note 5).

**Fig. 5:**
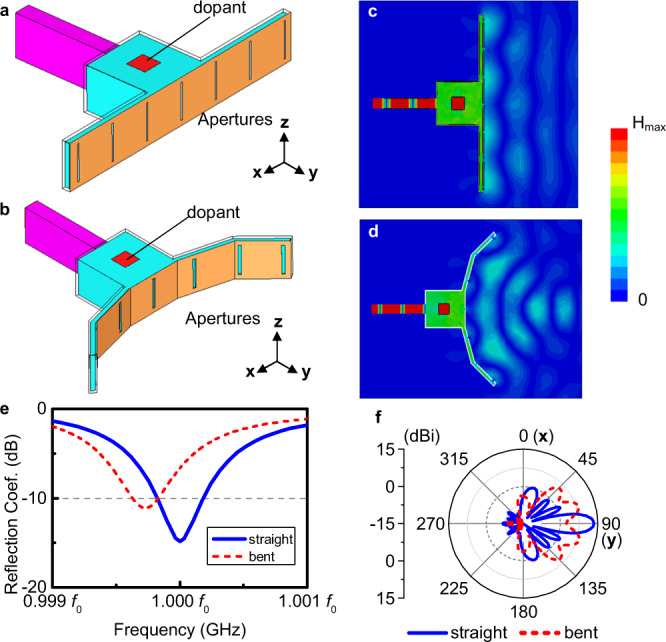
Flexible-wavefront radiator switching from directional beam to near-field focusing. **a** The 3D structure with a straight radiation aperture. **b** The 3D structure with a bent radiation aperture. **c**, **d** The time snapshots on the magnitude of magnetic fields at the central plane of these two antennas. **e** The simulation results for the reflection coefficients. **f** The angular distributions in the xy-plane.

Another application is the design of multi-functional antennas, for instance, changing the radiation directions from directional beaming to near field focusing. In these two extreme cases, wavefronts of the radiation have two completely different manners. To generate a directional radiation, a planar wavefront is demanded so that the magnitude and phase in the near field is the same at every point and the power is equally distributed. In contrast, the power is concentrated at a single point in the focusing scenario. Hence, it is usually challenging to design one antenna that embraces both functionalities. With the geometry-invariant properties discussed above, it becomes possible to shape the wavefront from a planar to a curved surface by bending the aperture, which changes a high gain beam to near field focusing without perturbating the operating frequency. The 3D geometric design is shown in the first two panels of Fig. 5, where we employ a T-shaped ENZ medium containing a rectangular cavity and an extended aperture. Seven slots are placed with even spacings on the aperture which we assume can be bent into different shapes. For the detailed geometries, please see Supplementary figs. 8 and 9 and the Supplementary Note 6. Figures 5a and 5b depicts two extreme states where the aperture is straight and bent, radiating a directional beam or focusing in the near field. The simulation for the near-field and far-field performances of these two cases are plotted in Fig. 5c, d, f. In addition, the operating frequency remains almost the same with a fractional frequency shift of 0.03% under this bending as shown in Fig. 5e, which is in accordance with the theory of the geometry-invariant radiation modes. For situations when the aperture is asymmetrically deformed, please see Supplementary fig. 10 and Supplementary Note 6.

## Discussion

In summary, we have demonstrated, theoretically and experimentally, that the operating frequency of an antenna composed of an ENZ medium is determined by the medium’s dispersion rather than its geometry. It has been demonstrated that for different antenna geometrical shapes the magnetic field inside the ENZ medium remains uniform at the ENZ frequency, and as a result, such change of shape of the ENZ medium only has impact on the wavefront and the angular distribution of the radiated fields, while leaving the operating frequency and the input impedance unchanged at the ENZ frequency. By carefully tuning the effective permeability of ENZ medium using photonic dopants, a perfect impedance matching from the source to free space is realized. With this enhanced radiation efficiency, we have examined the different geometry-independent antennas via both numerical and experimental methods on several prototypes with various sizes and shapes. Such a phenomenon may inspire various interesting applications, including a coded beam scanning scheme and a near/far-field wave-front manipulation via the geometry deformation.

## Methods

The numerical simulations in the main text and the supplementary materials are conducted with two different simulation softwares. The 2D simulation results are obtained using the COMSOL Multiphysics® 5.5. A rectangular port has been used for TEM wave incidence with the power of 1 W. The free space is truncated by a 200 × 200 mm^2^ rectangle and its boundaries are set as impedance boundaries in which the material is selected as air. For the calculation of the angular distributions of radiated power, a circle with a radius of 95 mm is used instead of the rectangle. The maximum size of the mesh element is 6.434 mm and the minimum size is 0.06 mm. Simulations on 3D structure have been carried out with CST Microwave Studio 2016. The frequency domain solver is adopted with tetrahedral meshes. A 50 Ohm discrete port is used for excitations at the position where the SMA connectors are located. The aluminum and copper used in the model are seen as perfect electric conductor (PEC) and their Ohmic losses are neglected. In particular, the aluminum shell of the ENZ cavity is set to be PEC material and the copper covering the waveguide is simulated by applying PEC boundaries on the Teflon brick. An air box is used with a distance of a quarter wavelength at 5.8 GHz (12.9 mm) to the simulated structure and the radiating boundaries are applied on the surface. The reflectance of the radiation boundary is lower than 1e-4.

## Supplementary information

Supplementary Information

## Data Availability

All the data and simulation files that support the findings of this study are both available at Dropbox repository (https://www.dropbox.com/sh/6nt7mah9vox6go3/AACp1gEBkenxZrd-n7Oca7_La?dl=0).
