# Supplementary material for: Geometry-independent antenna based on Epsilon-near-zero medium
Source: Nat Commun. 2022 Jun 22;13:3568. doi: 10.1038/s41467-022-31013-z (PMC9217913; doi:10.1038/s41467-022-31013-z)
Supplement: Supplementary file 1 — Supplementary Information [file 41467_2022_31013_MOESM1_ESM.pdf]

Geometry-Independent Antenna Radiation Based on Epsilon-Near-Zero Medium

Li et al.

Supplementary Figures

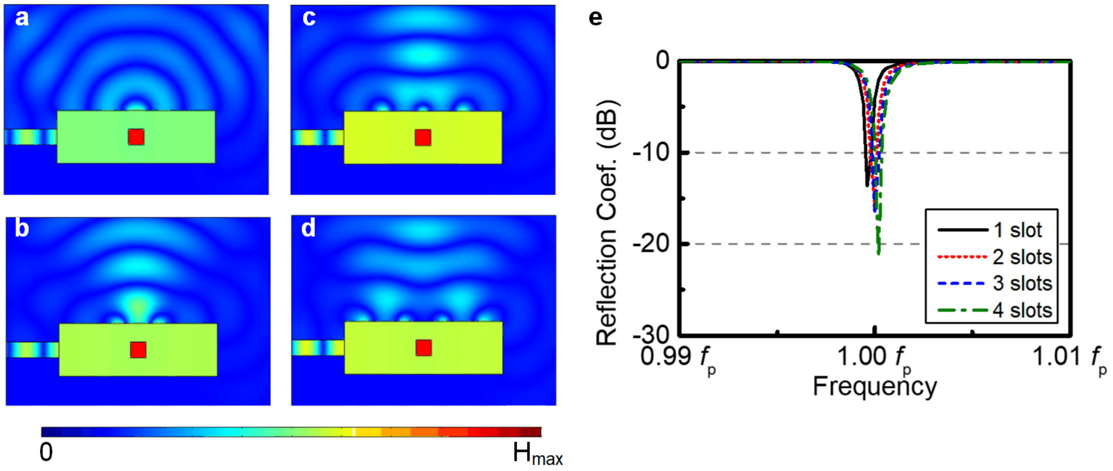

Supplementary Figure 1 | Simulation results for validation of the slots' impedance properties on 2D structures. **(a) to (d)** snapshots of the simulated magnetic fields of ENZ radiators with 1 to 4 slots. The ENZ host is 120×40 mm^2 and the width of each slot is 12 mm, 6 mm, 4 mm and 3 mm in each case. **(e)** Simulated reflection coefficients of antennas with 1 to 4 slots.

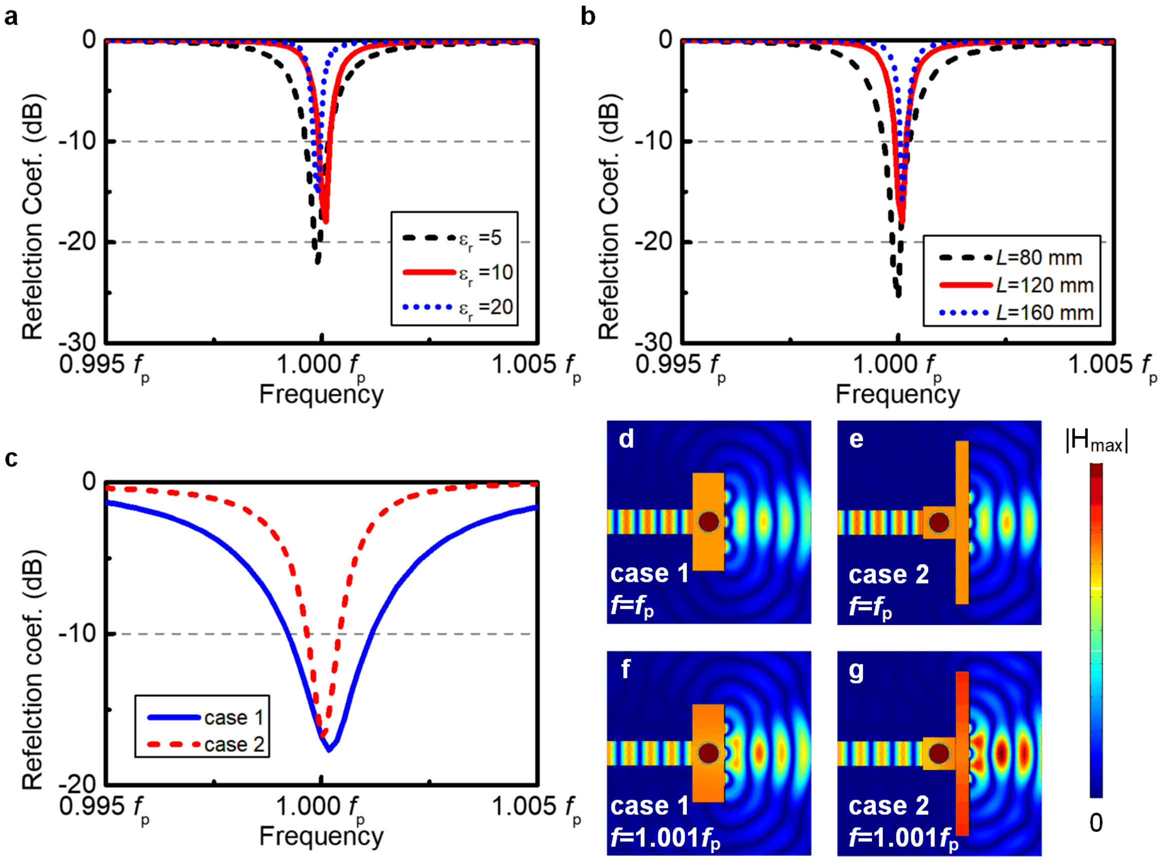

Supplementary Figure 2 | Discussions on bandwidths of the antenna with a relatively low Q factor. (**a**) Simulated reflection coefficients under different ε_d. The radius of the dopant is tuned to be 4.47 mm, 6.35 mm, and 9.08 mm when the relative permittivity is 5, 10, and 20. (**b**) Simulated reflection coefficients under different L, which is proportional to the area of the ENZ host. The radius of the dopant is tuned to be 6.38 mm, 6.35 mm, and 6.33 mm for each case and its permittivity is kept to be 10. (**c**) Simulated reflection coefficients of a low-Q ENZ antenna in two cases with different geometry shapes. In case 1, the geometry of it is 0.77×2.31 λ_0^2 (40×120 mm^2). In case 2, it is deformed into a T-shape composed by two rectangles of 0.77×0.77 λ_0^2 (40×40 mm^2) and 0.31×3.85 λ_0^2 (16×200 mm^2). (**d**) to (**g**) Snapshots on the magnitude of the magnetic fields at f_p and 1.001f_p for both cases.

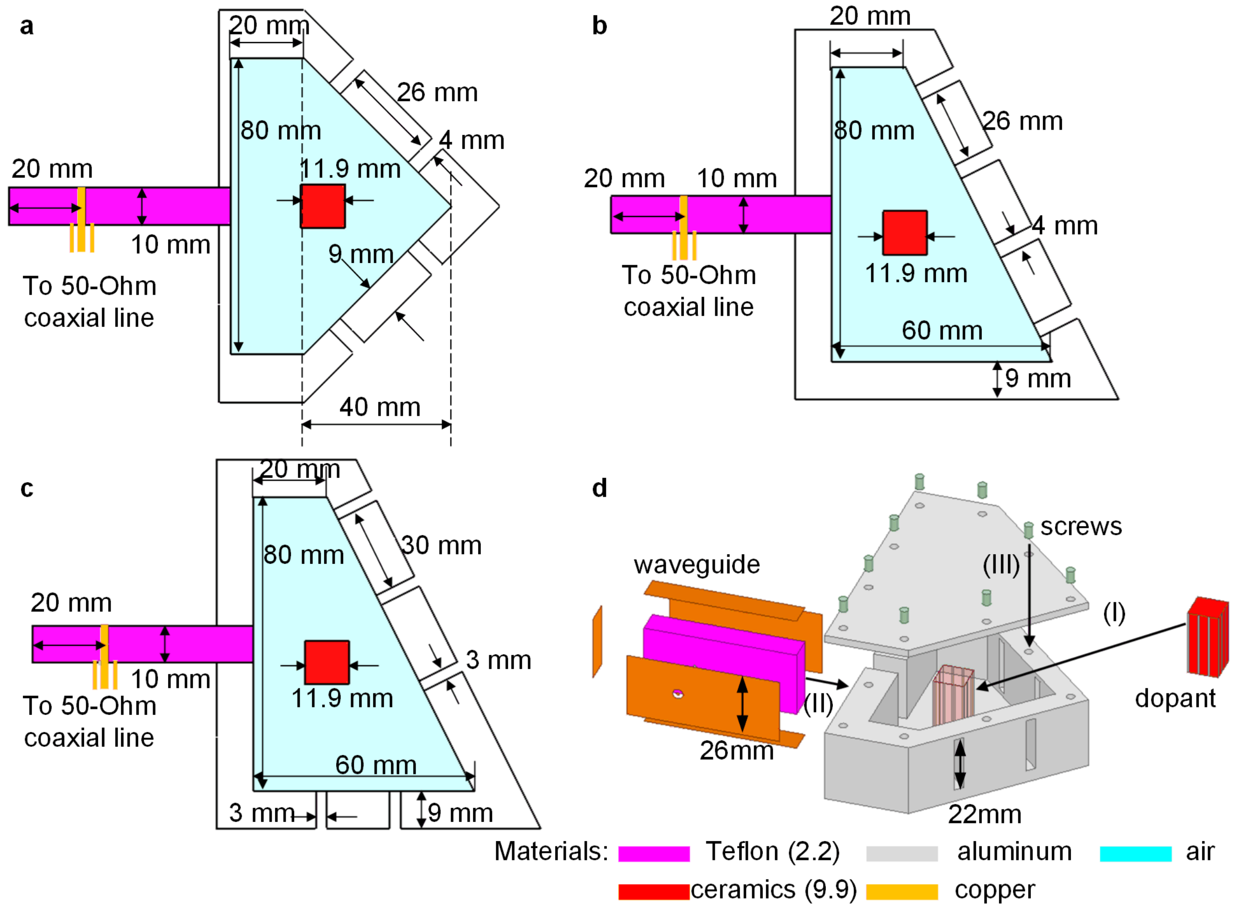

Supplementary Figure 3 | Geometry of the 3D structures used in the experiment. (**a** to **c**) Configurations in the central plane of Ant. 1 to 3. The detailed sizes are labelled on each panel. The relative permittivity of the Teflon used in these structures is 2.2 and that of the ceramic dopants is 9.9. All three antennas share a same cross section of the metal cavities. (**d**) The assembling process of Ant. 1. Firstly, the dopant printed with metal strips is placed in the cavity. Then, the Teflon brick coated with copper is inserted into the groove of the cavity's wall. Finally, a metal cover is screwed on the top of the cavity.

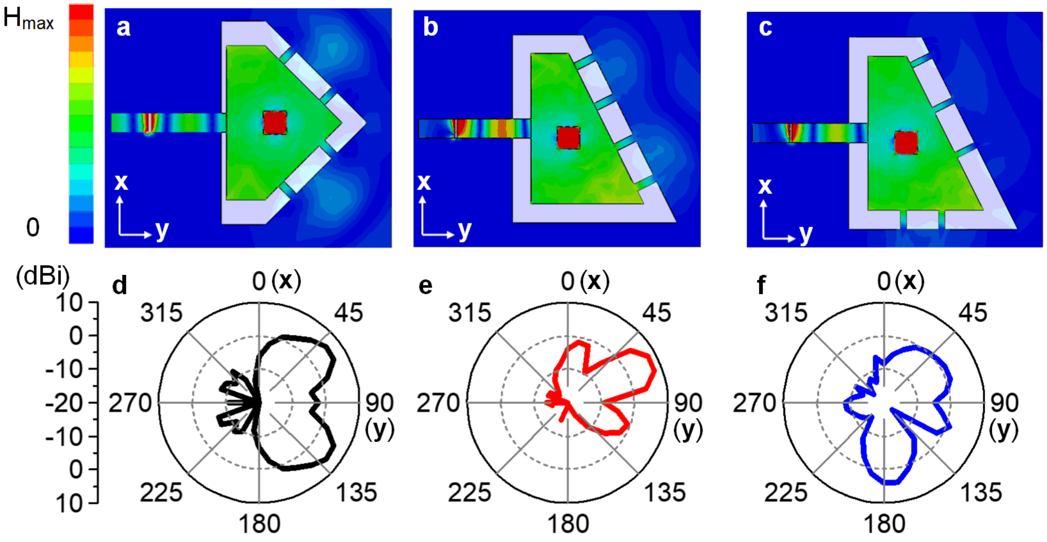

Supplementary Figure 4 | The simulation results for the fields and radiation patterns of the 3D antennas under radiating ENZ modes. (a to c) Magnitude of simulated magnetic fields in the central plane of Ant. 1 to 3. (d to f) simulation results for the gains at different directions within the xy plane corresponding to each antenna.

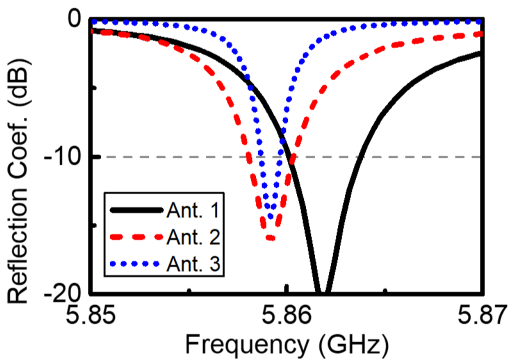

Supplementary Figure 5 | The simulation results for the reflection coefficients of the 3D antennas under radiating ENZ modes: A comparison of the simulated reflection coefficients of the three antennas, which are the same as those depicted in Figs. 3b, 3e, and 3h in the main text.

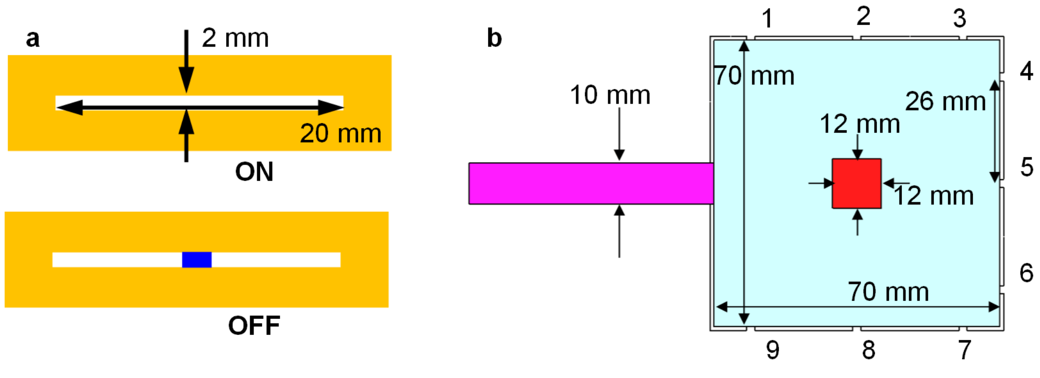

Supplementary Figure 6 | Geometry of the encoding beam antenna. (**a**) Sketch and geometry of the slot in the "ON" and "OFF" states, (**b**) Sketch and relevant dimensions of the whole antenna.

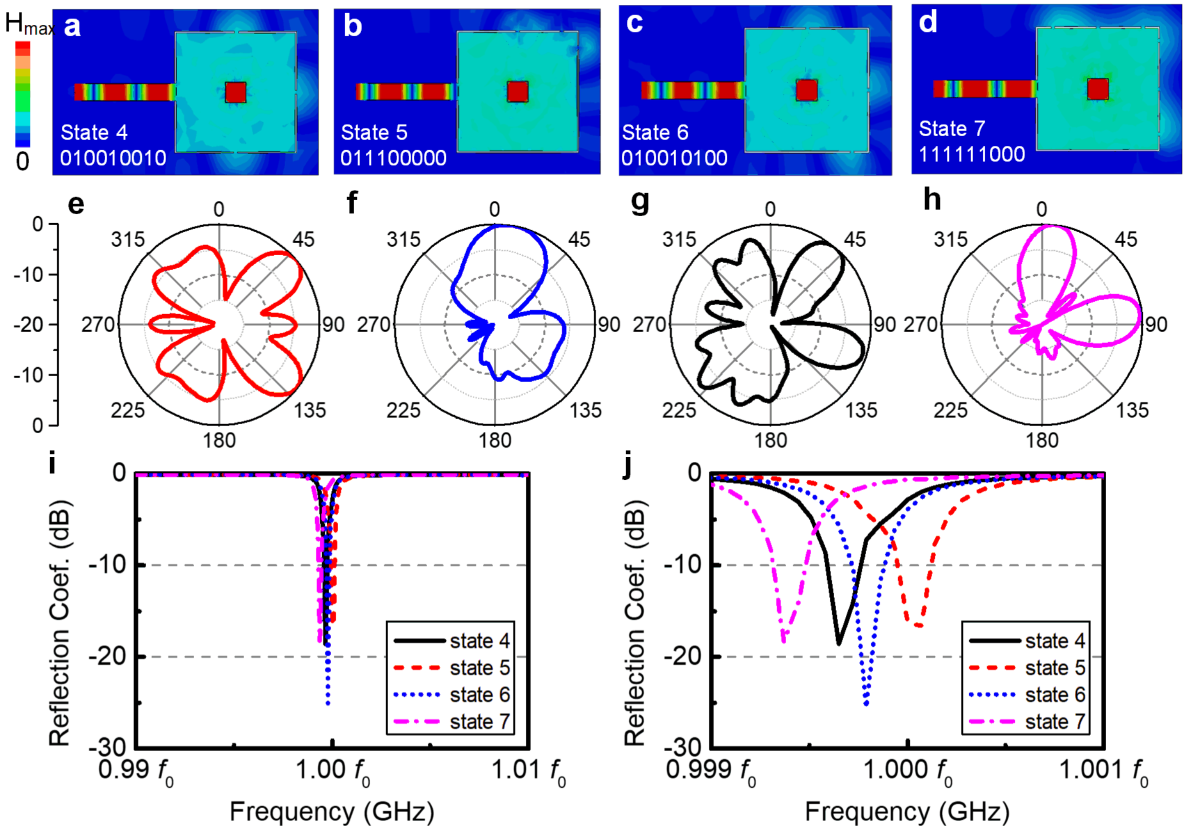

Supplementary Figure 7 | Additional states of the encoding beam antenna. (a)-(d) Time snapshots of magnetic fields of four additional states denoted as State 4-7 corresponding to codes "010010010", "011100000", "010010100", and "111111000", respectively. (**e**)-(**h**) The normalized radiation patterns of State 4-7, (**i**) the reflection coefficients of the four states with frequency varying from 0.99 f_0 to 1.01 f_0, and (**j**) a zoomed-in view of (i) from 0.999 f_0 to 1.001 f_0.

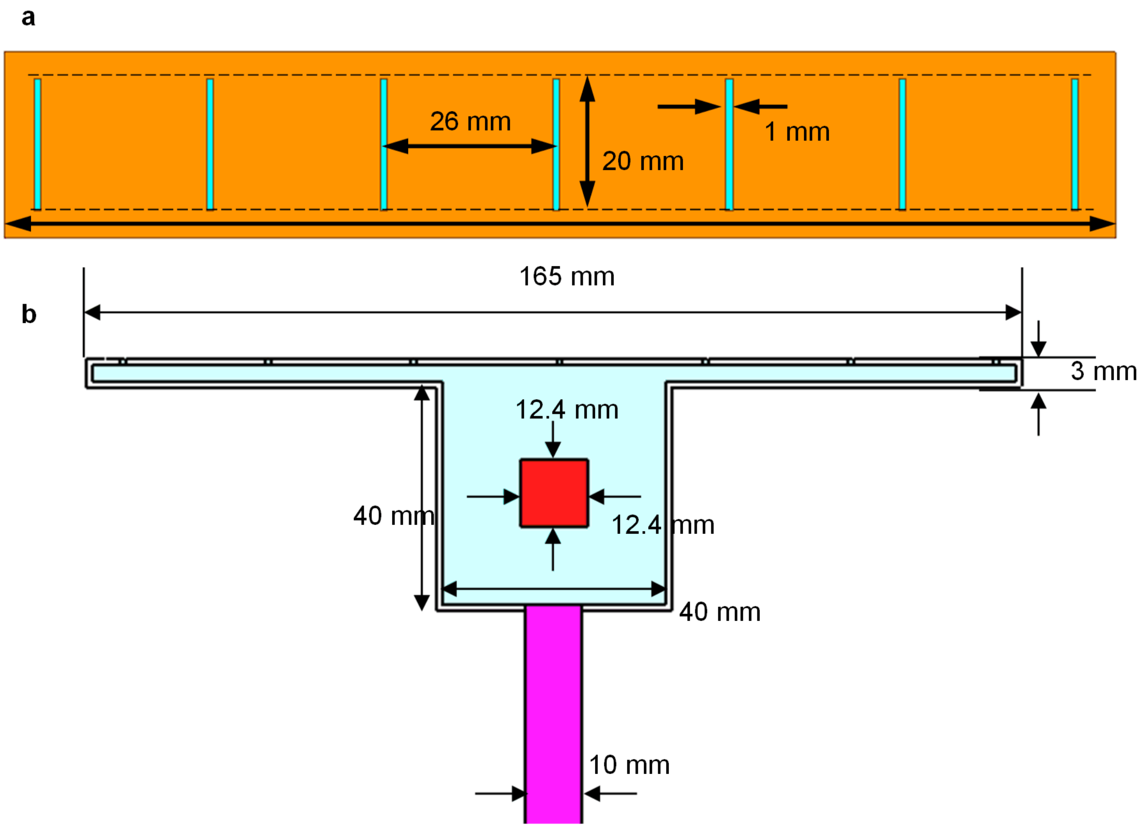

Supplementary Figure 8 | Geometry of the planar-wave state of the multi-functional antenna. (**a**) Sketch and geometry of the structure and relevant dimensions of the aperture from the top view and (**b**) of the multi-functional antenna from the side view. The sizes are labeled on the geometry.

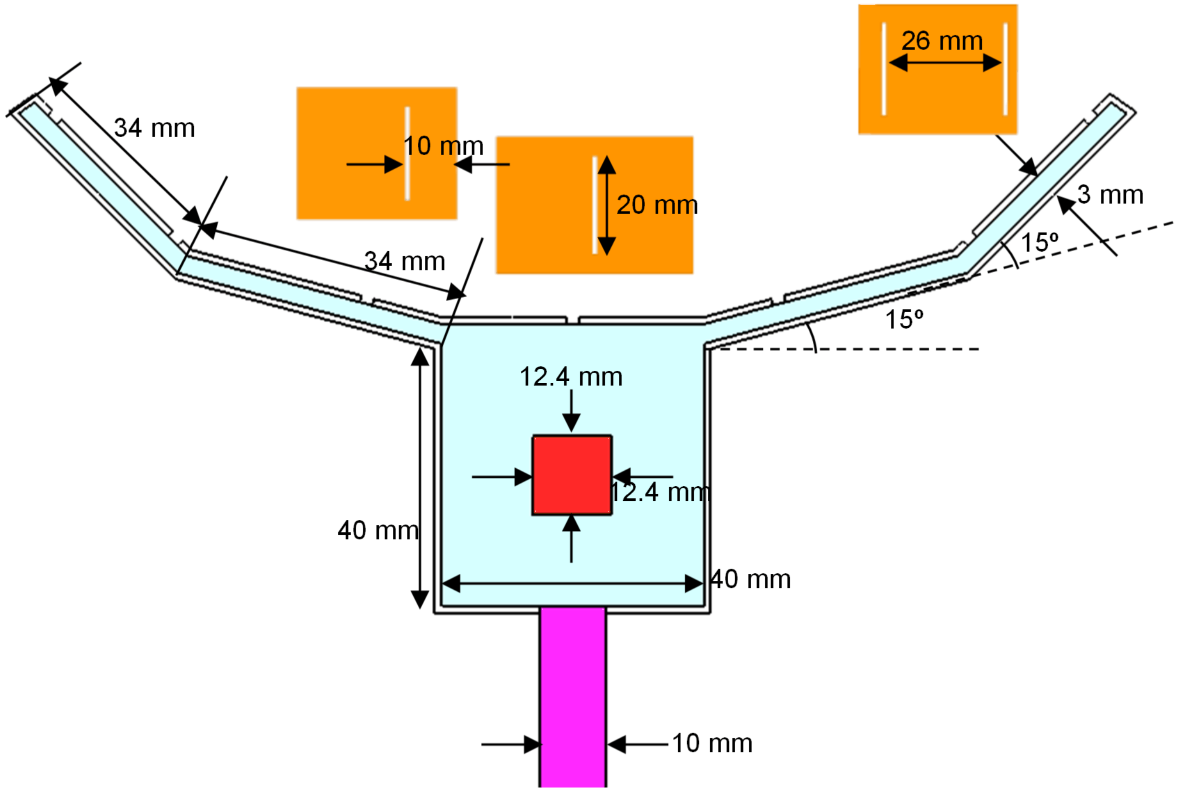

Supplementary Figure 9 | Geometry of the focusing state of the multi-functional antenna. Sketch of the geometry of the structure and relevant parameters of the bent-aperture state of the multi-functional antenna from the side view. The sizes are labeled on the geometry.

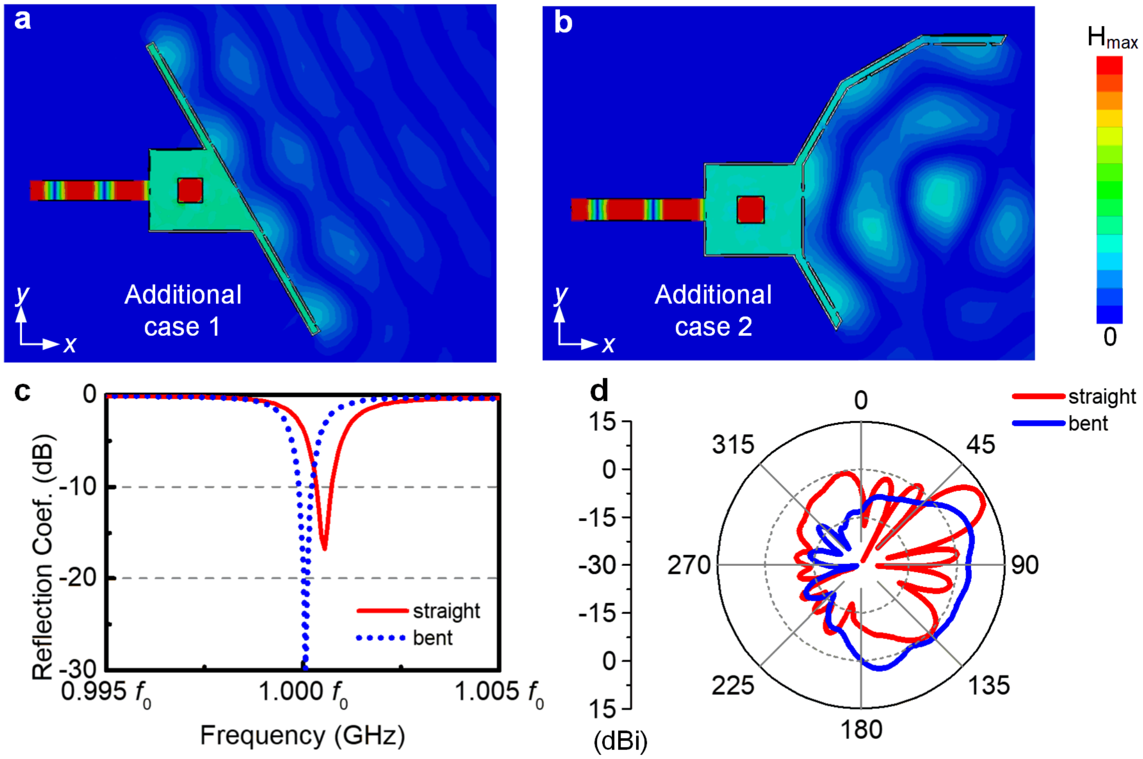

Supplementary Figure 10 | Asymmetric configurations of the flexible antenna example (a) simulation results for the magnetic field distributions of the straight aperture case when the aperture is tilted to 120°, (b) simulation results for the magnetic field distributions of the curved aperture case when the aperture is asymmetric, (c) the reflection coefficients, and (d) the radiation patterns.

Supplementary Note 1. Further discussion on the impedances of radiating apertures

In this Supplementary Note we investigate the impedance of a single radiation aperture. As defined in the main text, the 2D radiating impedance of the n-th aperture is $Z_n = R_n\text{-}iX_n= V_n/ H_0$ where V_n and H_0 are the voltage and magnetic fields on the aperture. Notice that the unit of the 2D impedance is Ω·m for it is defined by the ratio of voltage over the magnetic field. Here, some further quantitative discussions on Z_n of apertures in planar shapes are presented. We first neglect the edge effect and assume that the electric field is uniform over the aperture, from which we get $V_n=E_nl_n$. For an electrically large aperture, the ratio between the electric field and the magnetic field numerically equals to a constant that η_0=377 Ω which is the wave impedance in the free space because of the continuity of the fields on the aperture. For an aperture smaller than a wavelength, nevertheless, the expression that Re(E_n/H_0) = η_0 also holds as an approximation where Re[·] represents the real part operator. As a result, the real part of the 2D impedance of the aperture is Re(Z_n) = $\eta_0 l_n$.

When this approximation holds, the total 2D radiating resistance is calculated by $R_{total} = \sum_n R_n = \eta_0 \sum_n l_n$ when the mutual couplings between the apertures are weak enough to be neglected. This equation shows that the real part of the total impedance is only related to the total length of the apertures so that splitting a single aperture into several small apertures has little contributions to the variance of the frequency response. In addition to the real part, the capacitance of the aperture serving as a narrow gap between two conductors also generates the imaginary part X_n. Since X_n is negative, the relative permeability of the ENZ host is supposed to be positive to reach a perfect impedance matching. The existence of X_n may cause some frequency shift when a total-length-invariant splitting is performed on the aperture, however, the imaginary part X_n is usually very small compared to X_{ENZ} so that the reactance can be neglected. In addition, it is independent of the positions of the apertures. To verify that the total-length-invariant splitting of the aperture has little effects on the resonant frequencies and the reflection coefficients, a group of numerical simulations are launched. On an ENZ host of 2.31×0.77 λ_0^2 (120×40 mm^2), N slots are placed on a single side with the width of each to be (12/N) mm. A square dopant is utilized to tune the permeability of the ENZ medium[31,36] and the parameters are the same as Fig. 2 of the main text. The simulated results are depicted in Supplementary figure 1, demonstrating that the operating frequency is rarely changed by splitting a single slot into several slots while keeping the total length unchanged. This small difference in resonant frequency is because of the reactance in these four cases are not the same

while the difference in the magnitude of the reflection coefficients is mainly due to the mutual couplings and the error on calculating the impedance by using the approximation $\mathrm{Re}(E_n/H_0) = \eta_0$. Nevertheless, the reflection coefficients of all the cases are lower than -10 dB which indicates that more than 90% of the power is injected into the antenna rather than reflected back.

Supplementary Note 2. Theoretical analysis of impact of the waveguide channel length on the EMNZ resonance

The Q factor of a resonating mode is defined as the ratio of the energy stored in the resonant cavity to the energy dissipation per temporal period. All the energy discussed in the following are characterized by the energy per unit length in the out-of-plane direction, which has a unit of J/m. For a perfectly matched radiating mode, the energy radiated into free space per cycle is equal to that fed into the resonant cavity, which is calculated as $W_\mathrm{r} = Z_0 H_0^2/2$. As a result, the radiated energy is not related to the specific shape of the ENZ host. The energy stored inside the doped ENZ media is calculated by $W_\mathrm{s} = W_\mathrm{m,ENZ} + W_\mathrm{m,d} + W_\mathrm{e,d} + W_\mathrm{e,disp}$ where $W_\mathrm{m,ENZ}$, $W_\mathrm{m,d}$, and $W_\mathrm{e,d}$ denote respectively the magnetic energy stored in ENZ host, in the dopant and the electric energy in the dopant[43]. Since the ENZ host is dispersive, the electric energy stored in ENZ host is denoted as $W_{e,\mathrm{disp}} = \frac{1}{4}\iint_A (\partial\varepsilon / \partial\omega) E^2 dA$, which is described by a Drude model where the permittivity is $\varepsilon_\mathrm{h}(f) = 1 - f_\mathrm{p}^2/f^2$.

Since the Q factor is inversely correlated to the impedance bandwidth of the antenna, it is necessary to quantitatively investigate this important parameter. Here we calculate the energy terms $W_\mathrm{m,ENZ}$, $W_\mathrm{m,d}$, and $W_\mathrm{e,d}$. The cross-sectional shape of the dopant is chosen as a circle rather than a rectangular for the simplicity in the following derivation. In this case, the effective permeability of the doped ENZ medium is described by a simple equation[1]:

$$\mu_\mathrm{eff} = \frac{\mu_0}{A}\left[A - \pi r^2 + \pi r^2 \frac{2\,\mathrm{J}_1(k_d r)}{k_d r\,\mathrm{J}_0(k_d r)} \right] \tag{S1}$$

Furthermore, a closed form solution can be derived of the electric and magnetic energy stored in the ENZ host and the dopant. We first derive the field distributions inside the dopant. To calculate the stored energy $W_{m,\mathrm{ENZ}}$, $W_{m,\mathrm{d}}$, and $W_{e,\mathrm{d}}$, the field distributions are derived. By solving the wave equations $(\nabla^2 + k_d^2)\,\mathbf{H} = 0$ with the boundary condition $\mathbf{H} = \mathbf{H_0}$

in the dopant, it can be derived that the magnetic field within the dopant is

$$\mathbf{H}(\rho) = H_0 \frac{\mathrm{J}_0(k_d \rho)}{\mathrm{J}_0(k_d r)} \hat{\mathbf{z}} \tag{S2}$$

and the electric field is

$$\mathbf{E}(\rho) = \frac{H_0}{-i\omega\varepsilon_d} \frac{\mathrm{J}_1(k_d \rho)}{\mathrm{J}_0(k_d r)} \hat{\boldsymbol{\theta}} \tag{S3}$$

The stored magnetic energy in the dopant is calculated by

$$W_{\mathrm{m,d}} = \frac{1}{4}\iint_{A_d} H(\rho) B^*(\rho) = \frac{1}{4}\iint_{A_d} \mu_0 \left|H(\rho)\right|^2 \tag{S4}$$

which turns out to be

$$W_{\mathrm{m,d}} = \frac{\pi\mu_0 H_0^{\ 2}}{2\mathrm{J}_0^{\ 2}(k_d r)} \int_0^r \mathrm{J}_0^{\ 2}(k_d \rho) d\rho = \frac{\pi\mu_0 r^2 H_0^{\ 2}}{4\mathrm{J}_0^{\ 2}(k_d r)} \left[\mathrm{J}_0^{\ 2}(k_d r) + \mathrm{J}_1^{\ 2}(k_d r)\right] \tag{S5}$$

Similarly, the stored electric energy in the dopant is calculated by

$$W_{\mathrm{e,d}} = \frac{\pi\mu_0 H_0^{\ 2}}{2\mathrm{J}_0^{\ 2}(k_d r)} \int_0^r \mathrm{J}_1^{\ 2}(k_d \rho) d\rho = \frac{\pi\mu_0 r^2 H_0^{\ 2}}{4\mathrm{J}_0^{\ 2}(k_d r)} \left[\mathrm{J}_0^{\ 2}(k_d r) + \mathrm{J}_1^{\ 2}(k_d r) - \frac{2}{k_d r}\mathrm{J}_0(k_d r)\mathrm{J}_1(k_d r)\right] \tag{S6}$$

It can be observed from Eqs. (S5) and (S6) that both the area of the ENZ host and the permittivity of the dopant have considerable impacts on the Q factor by determining the stored energy W_s. This parametric study shows that the Q factor increases as the ENZ host's total area *A* and the dopant's permittivity ε_d become larger when the radius of the dopant is carefully tuned to maintain an unchanged resonant frequency. This happens for the reason that either a larger A or ε_d increases the ratio $\mathrm{J}_1(k_d r)/\mathrm{J}_0(k_d r)$ according to Eqs. (S5) and (S6). Since $\mathrm{J}_1(k_d r)/\mathrm{J}_0(k_d r) >> 1$ at the resonance of the radiating mode, the total stored energy W_s and the Q factor of the radiating mode both increases. For verification we launch two series of simulations on ENZ antennas with different areas and dopants. For the first group, we consider ENZ antennas with sizes of 1.54×0.77 λ_0^2 (80×40 mm^2), 2.31×0.77 λ_0^2 (120×40 mm^2), and 3.08×0.77 λ_0^2 (160×40 mm^2). Here the dopant's permittivity is kept the same of 10.0 while the radius is tuned to be 6.38 mm, 6.35 mm, and 6.33 mm for each case, ensuring a fixed resonance frequency at f_p. For the second group, the total size is kept to 2.31×0.77 λ_0^2 (120×40 mm^2) while three different dopants are used, which have permittivities of 5, 10, 20 and radii of 9.08 mm, 6.35 mm, and 4.47 mm, respectively. The simulated reflection coefficients are shown in Supplementary figures 2a and 2b for the first and second group, depicting that a narrower impedance bandwidth is detected in a larger ENZ cavity or with smaller dopants of higher permittivity. These results are in good agreement with the analytical ones. To lower the Q factor and enhance the bandwidth, we can thus shrink the size of the ENZ cavity and use low-permittivity dielectric inclusion with larger size as the dopant. In addition, enlarging the size of the radiating apertures also contributes to the decreasing of the Q factor because of a larger W_r. which seems to be a trivial conclusion.

With the parametric results gained above, we investigate an ENZ antenna with lowered Q factor by using a dopant shaped in a dielectric rod whose radius is 0.27 λ_0 (14.07 mm) and relative permittivity is 2.2. Each aperture of the three is enlarged to 10 mm while the thickness of the feeding waveguide also increases to 30 mm for impedance

matching. With these two major changes, the bandwidth of the antenna based on an ENZ cavity with total area of 1.78 λ_0^2 (4800 mm^2) is considerably enhanced. We numerically examined two specific cases, noted as Case 1 and Case 2, in which the antenna's geometry is deformed into a rectangle or a T-shape, respectively. In particular, the rectangular cavity in Case 1 has a geometry of 0.77×2.31 λ_0^2 (40×120 mm^2) while the T-shape used in Case 2 is constructed by combining two rectangles with sizes of 0.77×0.77 λ_0^2 (40×40 mm^2) and 0.31×3.85 λ_0^2 (16×200 mm^2). The simulated reflection coefficients are depicted in Supplementary figure 2c from which one can observe that unlike the geometry-independent resonance frequency, the antenna's bandwidth varies as the antenna is deformed, thus demonstrating a geometry-dependent Q factor. This phenomenon results from the fact that the magnetic field is not uniform at frequencies beyond f_p where the permittivity of the host medium is not zero. To further illustrate this issue, the snapshots of magnetic fields are shown in Supplementary figures2d to S2g at both f_p and 1.001f_p for these two cases. At exactly f_p, the magnetic field distributes strictly uniformly within the ENZ medium whatever shape it is. In contrast, the magnitude of the magnetic field is not uniform at 1.001 f_p especially at the place away from the port and dopant. As a result, the geometry-invariance is not kept at this frequency and the impedance matching is affected by the deformations. In addition, this is also due to the stored energy term $W_{e,\text{disp}} = \frac{1}{4}\iint_A (\partial\varepsilon / \partial\omega) E^2 dA$ which is caused by the material's dispersions. Since the electric field satisfies the equation that $\nabla \times \mathbf{E} = i\omega\mu_0\mathbf{H}_0$, the spatial variance of electric field does not vanish as that of the magnetic field does, resulting in a geometry-dependent $W_{\text{e,disp}}$ together with a Q factor varying with deformations.

Supplementary Note 3. Detailed configurations of the 3D structures used in experiments.

The geometries of the experimental platforms are shown in Supplementary figure 3. Take Antenna 1 for instance, as shown in Supplementary figure 3a, the antenna contains a pentagon-shaped 10-mm-thick metal cavity with a height of 26 mm and a cross section of 1600 mm^2. A dielectric block with a size of 11.9×11.9×26.0 mm^3 made by ceramics with permittivity of 9.9 is placed at any location inside the cavity as the dopant. 12 metal lines are printed on the four side planes evenly to suppress degenerated resonances. On two of the side walls of the cavity, four slots with the same size of 4×22 mm^2 are etched with an intercept of 26 mm. Another wall is slotted by a rectangular window of 10×26 mm^2, in which a waveguide is placed. The waveguide is constructed by Teflon (ε_r=2.2) brick coated with copper and a SMA coaxial connector is soldered on. The configurations of Ant. 2 and 3 are similar to that of Ant. 1. The only difference is the shape of the cavity and the positions of the slots. Detailed geometric parameters of other two antennas

are labelled on Supplementary figures 3b and 3c. These three structures are assembled in a same process depicted in Supplementary figure 3d in which the Ant. 1 is taken as an example.

Supplementary Note 4. Simulation results for the 3D structures used in the experiment.

With the use of commercial full-wave electromagnetic simulation software CST Microwave Studio, the three radiating ENZ resonating modes supported by Ant.1 to 3 are first numerically calculated and analyzed. The snapshots of magnetic fields at the central plane are shown in Supplementary figures 4a-4c. The magnetic field performs a unified magnitude and phase as in 2D ENZ media and their directions are all perpendicular to the central plane. An enhancement of the magnitude of the magnetic fields is also observed in each antenna as in the 2D structure. In addition, the reflection coefficients and angular distributions of the radiated power are plotted in Supplementary figures 4d-4f and Supplementary figure 5. The reflection coefficients are almost kept the same under variation of the geometric structure, while the wavefronts and the radiation directions are changed by the deformations. Small frequency shifts in the resonance frequencies are mainly due to the higher order modes such as TE_{30} or TE_{50} modes generated by mode mismatching on the slots, which are evanescent in the waveguide or cavity. Under the excitation of these modes, the waveguide is not exactly equivalent to ENZ medium. Nevertheless, the magnitudes of these higher modes are small enough and they attenuate in a short distance only contributing with a residual impedance loading.

Supplementary Note 5. Detailed geometrical parameters and additional states for the coding beam application example.

An antenna with nine-bit encoded radiation has been discussed in the main text. Here the detailed geometric structures are presented. As shown in Supplementary figure 6a, a slot is controlled to be either a radiating aperture or a conducting boundary simply by a switch in the center of the slot. When the switch is turned off, the slot is switched “ON” where it is capable for radiation. When the switch is turned off, the slot is switched “OFF” and it serves as a conducting boundary. The detailed sizes of the whole antenna are shown in Supplementary figure 6b. The height of the antenna is 26 mm so that the air-filled cavity is equivalent to ENZ medium under f_p=5.77 GHz. A dielectric dopant with a size

of 12×12×26 mm^3 is doped inside the ENZ host. The feeding waveguide is filled with dielectrics whose permittivity is 2.2.

In addition to the three states shown in the main text as examples, we also investigate numerically four more states “010010010”, “011100000”, “010010100”, and “111111000”. In these cases, the slots switched “ON” are distributed arbitrarily on either two or three sides of the cavity. The field distributions and radiation patterns of states 4-7 are plotted in supplementary figures 7a-7d from which one can observe that the magnetic field within the ENZ media has a uniform distribution. The radiation patterns are also depicted in supplementary figures 7e-7h. Since the radiators are arbitrarily selected, the radiation patterns are irregular. The reflections are plotted in supplementary figures 7i and 7j. The resonance frequency remains almost unchanged when compared with the reference one while the maximum frequency shift is smaller than 0.06%, validating the proposed assertion that the resonance frequency is indeed effectively independent of the selection of radiators. Similar to that in the main text, opening more slots may change the total radiation impedance and may introduce more reactance to the antenna, thus causing a larger frequency shift.

Supplementary Note 6. Detailed geometrical parameters and additional cases of the multi-functional antenna.

The geometry structure of the multi-functional antenna with planar wave emission is presented in Supplementary figure 7. The height of the antenna is 26 mm so that the air-filled cavity is equivalent to ENZ medium under f_p =5.77 GHz. As depicted in Supplementary figure 7b, the T-shaped ENZ host is doped with a dielectric dopant with a size of 12.4×12.4×26 mm^3. The relative permittivity of the dopant is 9.9. A waveguide filled with dielectrics whose relative permittivity is 2.2 is utilized for feeding. When the multi-functional antenna discussed in the main text is bent to realize a near field focusing, its geometry is configured into the one shown in Supplementary figure 8. The thin aperture of the T-shaped ENZ host is bent into five sections while the main part of it is not changed. The feeding waveguide and the dopant are kept the same.

In addition to the symmetric cases discussed in the main text, we also investigate numerically the cases where apertures are not symmetrically shaped. Specifically, two cases with either a straight aperture or a curved aperture have been investigated, as the asymmetric counterparts to the symmetric configurations discussed in the manuscript. The detailed

configuration together with the simulation results for the magnetic field of the additional case 1 is shown in supplementary figure 10a where the aperture is tilted to the 120° direction. From the field distribution one can observe a uniformly distributed magnetic field and a planar wave emission perpendicular to the aperture. Another case, denoted as the additional case 2, is configured as a curved aperture with asymmetric length on two sides of the cavity. The structure and the magnetic field distribution are both characterized numerically in supplementary figure 10b, showing that it can also focus the emitted wave in the near field, which is similar to that in the main text of the manuscript. The reflection coefficients for the additional cases 1 and 2 are plotted in supplementary figure 10c. From this panel we note that the resonance frequency stays almost the same with a fractional frequency shift smaller than 0.05%. Moreover, the radiation patterns are also evaluated through simulations and the results are depicted in supplementary figure 10d. The beams are steered to different angles due to the structural asymmetry, and the straight configuration has a higher directivity, as expected.

Supplementary References

1. Rappaport, T. S., Xing, Y., MacCartney, G. R., Molisch, A. F., Mellios, E., & Zhang, J., Overview of Millimeter Wave Communications for Fifth-Generation (5G) Wireless Networks—With a Focus on Propagation Models, *IEEE Trans. Antennas and Propag.*, **65**, 6213-6230 (2017).
2. Kostinski, A. B., & Boerner, W. M., On the foundations of radar polarimetry, *IEEE Trans. Antennas Propag.*, **34**, 1395-1404 (1986).
3. Cloude, S. R., & Pottier, E., A review of target decomposition theorems in radar polarimetry, *IEEE Trans. Geosci. Remote Sensing*, **34**, 498-518 (1996).
4. Poon, A. S. Y., O'Driscoll, S., & Meng, T. H., Optimal Frequency for Wireless Power Transmission Into Dispersive Tissue, *IEEE Trans. Antennas and Propag.*, **58**, 1739-1750 (2010).
5. Liu, C., Guo, Y., Sun H., & Xiao, S., Design and Safety Considerations of an Implantable Rectenna for Far-Field Wireless Power Transfer, *IEEE Trans. Antennas and Propag.*, **62**, 5798-5806 (2014).
6. Pozar, D. M., *Microwave Engineering*, 3rd ed. New York: Wiley (2005).
7. Chu, L. J., Physical limitations on omni-directional antennas, *J. Appl. Phys.*, **19**, 1163-1175 (1948).
8. Nan, T., Lin, H., Gao, Y., et al. Acoustically actuated ultra-compact NEMS magnetoelectric antennas. *Nat. Commun.*, **8**, 296 (2017).
9. Tang, L., Kocabas, S., Latif, S., et al. Nanometre-scale germanium photodetector enhanced by a near-infrared dipole antenna. *Nat. Photonics* **2**, 226–229 (2008).
10. Zhu, W., Xu, T., Wang, H., Zhang, C., Deotare, P. B., Agrawal, A., & Lezec, H. J., Surface plasmon polariton laser based on a metallic trench Fabry-Perot resonator. *Sci. Adv.* **3**, e1700909 (2017).
11. Sorger, V., Oulton, R., Yao, J., Bartal, G., & Zhang, X., Plasmonic Fabry-Pérot Nanocavity, *Nano Lett.* **9**, 3489–

3493 (2009).
12. Yao, J., et al., “Three-dimensional nanometer scale optical cavities of indefinite medium,” *PNAS* **108**, 11327–11331 (2011).
13. Engheta, N., Pursuing near-zero response. *Science* **340**, 286–287 (2013).
14. Liberal, I., & Engheta, N., Near-zero refractive index photonics, *Nature Photon*, **11**, 149–158 (2017)
15. Maas, R., Parsons, J., Engheta, N., et al., Experimental realization of an epsilon-near-zero metamaterial at visible wavelengths. *Nat. Photonics* **7**, 907–912 (2013).
16. Chaimool, S., Rakluea, C., & Akkaraekthalin, P., Mu-near-zero metasurface for microstrip-fed slot antennas. *Appl. Phys. A* **112**, 669–675 (2013).
17. Mahmoud, A., & Engheta, N., Wave–matter interactions in epsilon-and-mu-near-zero structures. *Nat. Commun.*, **5**, 5638 (2014).
18. Silveirinha, M., & Engheta, N., Tunneling of Electromagnetic Energy Through Subwavelength Channels and Bends Using Epsilon-Near-Zero Materials, Phys. Rev. Lett., 97,157403 (2006).
19. Edwards, B., Alù, A., Young, M. E., Silveirinha, M. G., & Engheta, N., Experimental Verification of Epsilon-Near-Zero Metamaterial Coupling and Energy Squeezing Using a Microwave Waveguide, *Phys. Rev. Lett.* **100**, 033903 (2008).
20. Liu, R., Cheng, Q., Hand, T., Mock, J. J., Cui, T. J., Cummer, S. A., & Smith, D. R., Experimental Demonstration of Electromagnetic Tunneling Through an Epsilon-Near-Zero Metamaterial at Microwave Frequencies, *Phys. Rev. Lett.* **100**, 023903 (2008).
21. Alù, A., Silveirinha, M. G., Salandrino, A., & Engheta, N., Epsilon-near-zero metamaterials and electromagnetic sources: Tailoring the radiation phase pattern, *Phys. Rev. B* **75**, 155410 (2007).
22. Zhou B., & Cui, T. J., Directivity Enhancement to Vivaldi Antennas Using Compactly Anisotropic Zero-Index Metamaterials," *IEEE Antennas Wireless Propag. Lett.*, **10**, 326-329 (2011)
23. Enoch, S., Tayeb, G., Sabouroux, P., Guérin, N., & Vincent, P., A metamaterial for directive emission, *Phys. Rev. Lett.* **89**, 213902 (2002)
24. Jiang, Z. H., Wu, Q., Brocker, D. E., Sieber, P. E., & Werner, D. H., A Low-Profile High-Gain Substrate-Integrated Waveguide Slot Antenna Enabled by an Ultrathin Anisotropic Zero-Index Metamaterial Coating, *IEEE Trans. Antennas and Propag.*, **62**, 1173-1184 (2014).
25. Forati, E., Hanson, G. W., & Sievenpiper, D. F., An Epsilon-Near-Zero Total-Internal-Reflection Metamaterial Antenna, *IEEE Trans. Antennas and Propag.*, **63**, 1909-1916 (2015)
26. Suchowski, H., et al. Phase mismatch—free nonlinear propagation in optical zero index materials, *Science*, **342**, 1223–1226 (2013).
27. Powell, D. A., et al. Nonlinear control of tunneling through an epsilon-near-zero channel, *Phys. Rev. B* **79**, 245135 (2009).
28. Argyropoulos, C., D’Aguanno, G., & Alù, A., Giant second-harmonic generation efficiency and ideal phase matching with a double ε-near-zero cross-slit metamaterial. *Phys. Rev. B* **89**, 235401 (2014).
29. Silveirinha, M. G., Trapping light in open plasmonic nanostructures. *Phys. Rev. A* **89**, 023813 (2014).

30. Alù, A., & Engheta, N., All optical metamaterial circuit board at the nanoscale. *Phys. Rev. Lett.* **103**, 143902 (2009).
31. Liberal, I., Mahmoud, A. M., Li, Y., Edwards, B., & Engheta, N., Photonic doping of epsilon-near-zero media, *Science* **355**, 1058 (2017).
32. Silveirinha, M., & Engheta, N., Design of matched zero-index metamaterials using nonmagnetic inclusions in epsilon-near-zero media, *Phys. Rev. B*. **75**, 075119 (2007).
33. Nguyen, V., Chen, L., & Halterman, K., Total transmission and total reflection by zero index metamaterials with defects. *Phys. Rev. Lett.*, **105**, 233908 (2010).
34. Xu, Y., & Chen, H., Total reflection and transmission by epsilon-near-zero metamaterials with defects, *Appl. Phys. Lett.* **98**, 113501 (2011).
35. Liberal, I., Mahmoud, A. M., & Engheta, N., Geometry invariant resonant cavities, *Nat. Commun.* **7**, 10989 (2016).
36. Zhou, Z., Li, Y., Li, H., Sun, W., Liberal, I., & Engheta, N., Substrate-integrated photonic doping for near-zero-index devices, *Nat. Commun.* **10**, 4132 (2019).
37. Zhou, Z., Li, Y., Nahvi, E., Li, H., He, Y., Liberal, I., & Engheta, N., General impedance matching via doped epsilon-near-zero media, *Phys. Rev. Applied*, **13**, 034005 (2020).
38. Zhou, Z., and Li, Y., "Effective Epsilon-Near-Zero (ENZ) Antenna Based on Transverse Cutoff Mode," *IEEE Trans. Antennas Propag.*, **67**, 2289-2297, (2019).
39. Zhou, Z., and Li, Y., "An ENZ-Inspired Antenna with Controllable Double-Difference Radiation Pattern", *International Symposium on Antenna and Propagation, Xi'an, China*, (2019).
40. Li, Y., Liberal, I., Giovampaola, C. D., & Engheta, N., Waveguide metatronics: lumped circuitry based on structural dispersion. *Sci. Adv.* **2**, e1501790 (2016).
41. Edwards, B., & Engheta, N., Experimental verification of displacement-current conduits in metamaterials-inspired optical circuitry. *Phys. Rev. Lett.* **108**, 193902 (2012).
42. Qin, Y., Zhang, C., Zhu, Y., Hu, X., & Zhao, G., Wave-Front Engineering by Huygens-Fresnel Principle for Nonlinear Optical Interactions in Domain Engineered Structures, *Phys. Rev. Lett.*, **100**, 063902 (2008).
43. Zhang, K., & Li, D., *Electromagnetic Theory for Microwaves and Optoelectronics*, Springer, Berlin, (2007).
